# The Translational Data Catalog - discoverable biomedical datasets

**DOI:** 10.1038/s41597-023-02258-0

**Published:** 2023-07-20

**Authors:** Danielle Welter, Philippe Rocca-Serra, Valentin Grouès, Nirmeen Sallam, François Ancien, Abetare Shabani, Saeideh Asariardakani, Pinar Alper, Soumyabrata Ghosh, Tony Burdett, Susanna-Assunta Sansone, Wei Gu, Venkata Satagopam

**Affiliations:** 1 Luxembourg Centre for Systems Biomedicine, ELIXIR Luxembourg, University of Luxembourg, L-4367 Belval, Luxembourg; 2 Luxembourg National Data Service (PNED G.I.E), 6 avenue des Hauts-Fourneaux, L-4362 Esch-sur-Alzette, Luxembourg; 3 Oxford e-Research Centre, Department of Engineering Science, University of Oxford, 7 Keble Road, OX13QG Oxford, UK; 4 AstraZeneca, Data Office, Data Science & AI unit R&D, 136 Hills Rd, Cambridge, UK; 5 European Molecular Biology Laboratory, European Bioinformatics Institute (EMBL-EBI), Hinxton, CB10 1SD UK; 6 Frankfurt Institute for Advanced Studies (FIAS), Ruth-Moufang-Straße 1, D-60438 Frankfurt am Main, Germany

**Keywords:** Research data, Data publication and archiving, Data integration

## Abstract

The discoverability of datasets resulting from the diverse range of translational and biomedical projects remains sporadic. It is especially difficult for datasets emerging from pre-competitive projects, often due to the legal constraints of data-sharing agreements, and the different priorities of the private and public sectors. The Translational Data Catalog is a single discovery point for the projects and datasets produced by a number of major research programmes funded by the European Commission. Funded by and rooted in a number of these European private-public partnership projects, the Data Catalog is built on FAIR-enabling community standards, and its mission is to ensure that datasets are findable and accessible by machines. Here we present its creation, content, value and adoption, as well as the next steps for sustainability within the ELIXIR ecosystem.

## Introduction

Large multi-national research funders such as the Innovative Medicines Initiative (IMI, https://www.imi.europa.eu) and its successor, the Innovative Health Initiative (IHI, https://www.ihi.europa.eu/) bring together academics, major pharmaceutical companies, and information and service companies in the life sciences with the goal of driving health research and innovation, and translating them into tangible benefits for patients and society. With their multi-billion euro budgets, these initiatives are the world’s biggest public-private partnerships in the life sciences, funding a diverse range of projects across a number of priority disease areas, where safe, effective treatments are lacking, or where the impact on public health is expected to be the most substantial. However, despite the initiatives’ ambition of bridging the gap between key players in healthcare research, including universities, the pharmaceutical industries, regulators and other organisations that often work in isolation from each other, there is not yet any coordinated effort to track and record what types of data are generated within the funded projects. As a result, dissemination, deposition and interconnection of these project outputs remain sporadic and difficult, resulting in lost opportunities for data reuse and the danger of duplicating efforts.

Findability of scientific data and metadata is one of the cornerstones of the FAIR principles^1^. The principle attempts to address and redress the shocking cost^2^, both scientific and financial, of undiscoverable and non-persistent research data. Clinical and translational research, in particular, suffers from a lack of data availability due to the sensitive and confidential nature of clinical datasets and the legal constraints of data-sharing agreements, which often disincentivise researchers from sharing even high-level aggregated metadata outside the limits of a project partnership.

The predecessor of the Translational Data Catalog (Data Catalog for short) was developed in a partnership between the eTRIKS project (http://www.imi.europa.eu/projects-results/project-factsheets/etriks), an IMI infrastructure project, and the Luxembourg Node (https://elixir-luxembourg.org) of ELIXIR, an intergovernmental organisation that brings together life science resources from across. Launched in 2017, the Data Catalog was created as a ‘one-stop shop’ system to enhance the discoverability of the datasets generated and curated by, or reused in IMI research projects, as well as other translational H2020 research projects. The Data Catalog allowed partners to promote their solutions and encourage data sharing. The initial prototype was pivotal in showing the value of a metadata index to enhance the findability of these translational datasets, and foster more research collaboration between public and private organisations.

Since 2019, the Data Catalog has been reviewed and extended as part of the FAIRplus (https://fairplus-project.eu), another infrastructure project of the IMI programme. The enhanced version provides high-level metadata, such as project descriptions and contacts, data types and experiment types, on all projects funded by IMI. This latest iteration of the Data Catalog presents a unique collection of projects and datasets, covering a range of data types, leveraging semantic technologies to enable data discovery through the implementation of community standards and FAIR data principles. In this paper, we present the Data Catalog’s new data model, its underlying infrastructure and functionality, the improved content to support FAIRness, as well as its role and sustainability in the ELIXIR ecosystem.

## Results

### Data model: Building on existing community standards

The initial Data Catalog data model was a bespoke resource-specific model heavily skewed towards translational medicine and classic human clinical trial datasets, focusing on cohort and patient characteristics, treatments and interventions. This model made it difficult, however, to include thematically related study types such as pharmacological, toxicological or drug repurposing studies in animals or *in vitro* systems, which represent a large number of studies within IMI projects.

**Fig. 1:**
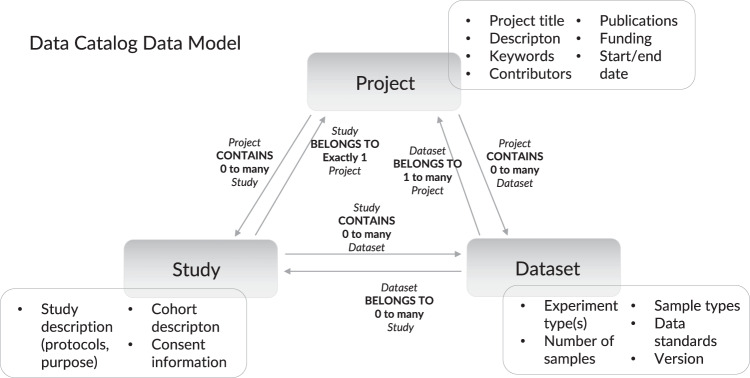
The Data Catalog Data Model. The model links the core data entities - Project, Study and Dataset - via directional relationships. Each core entity contains a set of relevant properties.

In order to widen the scope, as well as conform to a recognised community standard as recommended by the FAIR principles, the DAta Tag Suite (DATS)^3^ data model was adopted and adapted for use in the current version of the Data Catalog, an overview of which is presented in Fig. 1. DATS is a data description model designed and produced to describe datasets being ingested in DataMed^4^, a prototype for data discovery developed as part of the USA NIH Big Data to Knowledge and Data Commons programmes^5^. DATS was used to uniformly represent metadata across a number of projects, including the Genotype-Tissue Expression project (GTEx, https://www.gtexportal.org/home/) and Trans-Omics for Precision Medicine (TOPMed, https://www.nhlbi.nih.gov/science/trans-omics-precision-medicine-topmed-program). DATS is semantically compatible with the Data Catalog Vocabulary (DCAT)^6^, a Resource Description Framework (RDF) vocabulary designed to facilitate interoperability among data catalogues published on the web, as well as schema.org (SDO, https://schema.org/), which is a community-driven effort with a similar interoperability goal to DCAT but a more general-purpose scope. Many DATS properties have also been mapped to Open Biological and Biomedical Ontology (OBO) Foundry^7^ ontologies to further enhance the model’s semantic interoperability.

The structure of the Data Catalog DATS model is not dissimilar to the ISA (Investigation, Study, Assay) model^8,9^ but has greater flexibility as datasets can exist independently of studies, whereas ISA assays require the presence of a study in a data object. Synthetic datasets or knowledge graphs assembled from multiple datasets and published as a new dataset for example may not have any relevant study-level metadata, so being able to bypass the study level simplifies data representation.

Another key difference between the DATS and ISA model is their ultimate focus: while ISA is designed to present metadata about the experimental process, the measurements and tests involved, DATS focuses on different aspects of datasets, such as their availability, access restrictions and technical details. Metadata on experimental procedures is included to aid findability and interoperability, rather than in order to represent the full scope of the experiment.

While several commonly occurring metadata elements, such as “project website”, were added as explicit properties to the model, DATS remains very lightweight and flexible, enabling the representation of diverse data types and their respective characteristics in a consistent and interoperable manner.

### (Meta)data: Populating the data catalog

The content of the Data Catalog was generated from a range of different sources: At Dataset level, it consists of datasets generated from the projects FAIRified^10^ by FAIRplus as well as public datasets that have been curated and used in other IMI projects (e.g. a subset of datasets from NCBI’s Gene Expression Omnibus^11^ (GEO) curated by the IMI-eTRIKS^3^ project). At Project level, project information (metadata within the “Project” component of the extended DATS model) of all approved IMI projects has been catalogued by parsing and curating selected content from the “Project Factsheets” of the IMI website (http://www.imi.europa.eu/projects-results/project-factsheets). Besides the IMI projects, in line with the new data model, it is also possible to accommodate projects from other initiatives, such as Horizon 2020, as well as multiple datasets and studies for a given project, as illustrated in the entries for the COVIRNA (https://covirna.eu/) and SYSCID (https://syscid.eu/) projects, among others.

There is an ongoing effort to further curate all the metadata in the Data Catalog, including adding ontology annotations where possible, for concepts such as disease, data type, sample type, sample source and experiment type. Thanks to the DATS model’s flexibility, it is also possible to consistently encode ad hoc data quantifiers or categories particular to a specific study, dataset or data type. An example of this is illustrated in Table 1.

**Table 1:** Example characteristics for a study cohort.

Characteristic dimension	Dimension mapping	Value category	Category mapping	Dimension unit	Unit mapping
Age at enrollment	NCIT:C164338	Minimal value	SIO:001113	year	UO:0000036
		Maximal value	SIO:001114		
Weight	NCIT:C25208	Minimal value	SIO:001113	kg	UO:0000009
		Maximal value	SIO:001114		
Height	NCIT:C25347	Minimal value	SIO:001113	meter	UO:0000008
		Maximal value	SIO:001114		
BMI	NCIT:C16358	Minimal value	SIO:001113	kg/m2	UO:0000086
		Maximal value	SIO:001114		

Each characteristic consists of a high-level dimension, a set of value categories, each associated with one or more values, and a unit where appropriate. Each level is mapped to an appropriate ontology term.

The Data Catalog team engages actively with IMI projects, both closed and ongoing, for which currently only project-level metadata is available, to gather study and dataset-level metadata. We also regularly populate metadata for new projects as part of our data-hosting service at the ELIXIR Luxembourg Node (https://elixir-luxembourg.org/services/). In this context, the Data Catalog serves as both a discovery point and the user portal for requesting access to hosted data.

### Bioschemas integration: Small markup for great findability

In order to improve the findability of Data Catalog content through standard web searches, we integrated Bioschemas^12^ markup in all aspects of the Catalog. This markup, which is an extension of schema.org, is indexable by search engines and other services. Bioschemas is a flagship development of the ELIXIR Interoperability Platform and is being widely adopted in life science resources, aiming to increase their FAIRness along the findability axis. The FAIR Cookbook^13^ (https://faircookbook.elixir-europe.org/) includes recipes regarding search engine optimisation using Bioschemas (https://w3id.org/faircookbook/FCB010), which served as the basis for the implementation of the “DataCatalog” and “Dataset” profiles. Further profiles currently under development by Bioschemas may be integrated in the future.

### DATS export and data download: Making the content directly reusable

In order to facilitate the interoperability of the Data Catalog metadata with other metadata, as well as its import into other resources, the Data Catalog provides an export functionality that allows users to export all Data Catalog data as JSON files conforming to the DATS data model.

There is also a “Acces data” option, which was extended from the original Data Catalog, to give users direct access to data in cases where no special data access permissions are required, e.g. for publicly available datasets. For datasets with data access restrictions, the access restrictions in the Catalog entries are pulled from our Data Information System (DAISY)^14^ if the data are also hosted by ELIXIR Luxembourg. When datasets are hosted by external repositories, we encourage the encoding of the restrictions using Data Use Ontology^15^ (DUO) codes and licensing information where available, although we do not provide data access request functionality for these datasets. A “Request data access” option is only added to ELIXIR Luxembourg-hosted datasets. This is associated with a newly implemented login functionality linking to several identity providers such as the LifeScienceRI and ORCID. This function enables the integration with data access management systems such as the Resource Entitlement Management System (REMS, https://github.com/CSCfi/rems) used by ELIXIR Luxembourg. A user dashboard allowing identified users to access content that they have already been approved for is under development.

### FAIRification results: Showcasing FAIR datasets

**Fig. 2:**
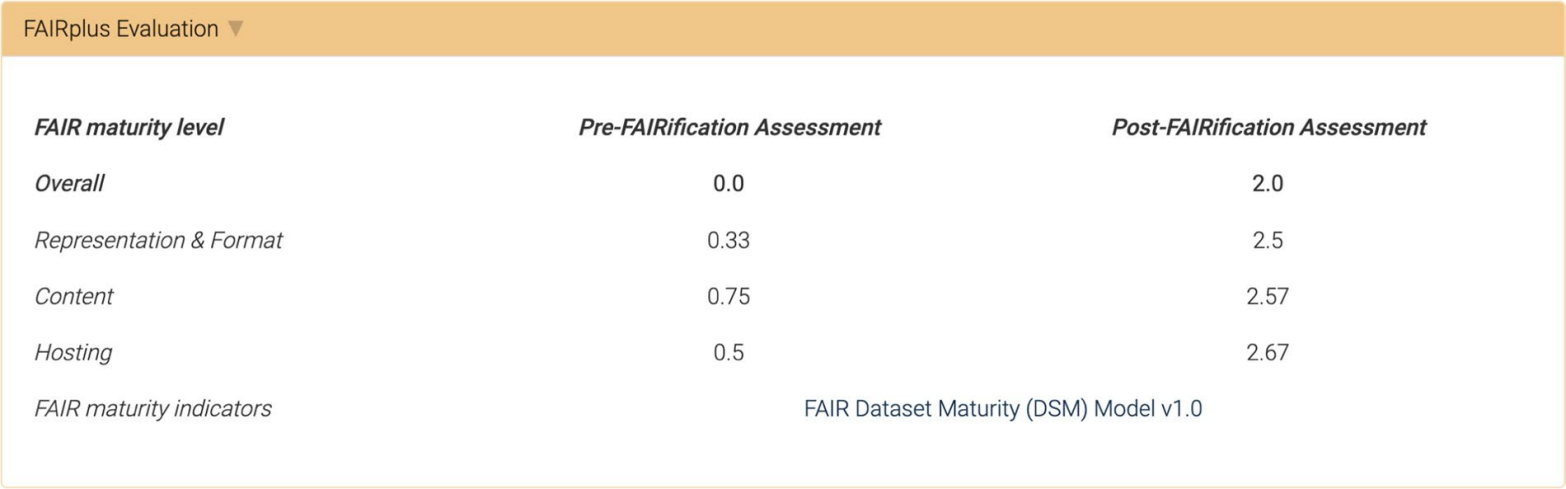
An example of a FAIRplus Evaluation result. The FAIRplus Evaluation panel lists the evaluation method used and the evaluation results, as well as links to the full assessment and the dataset (if publicly available).

All datasets FAIRified as part of the FAIRplus project are highlighted in the Data Catalog through a “FAIRplus Evaluated” badge displayed in the general datasets list, search results and on the page for the dataset (see Fig. 4), as well as for the study and project associated with the dataset. On the dataset page, a dedicated tab summarises the outcome of the FAIR maturity assessment with links to the full assessment and the data used to perform it (see Fig. 2).

### Usage statistics

The new Data Catalog website was released on 01/02/2021 and data analytics show both greater traffic and more user interactions in the first 18 months after its release (01/02/2021–31/08/2022) compared to the preceding 18 months (06/07/2019–31/01/2021). During this period, the analytics server logged a 139% increase in traffic, from 189 visits to 452, an average of about 25 per month. Visits originated from 29 different countries, with the majority of visits from Europe (412), followed by North America (19), Asia (18), South America (2) and Africa (1). A full list of usage statistics is available in Table 2.

**Table 2:** Usage statistics for the Translational Data Catalog for the 18 months before and after the release of the new web UI, gathered by the internal Matomo analytics server.

Metric	18 months after release *(01/02/2021–31/08/2022)*	18 months before release *(06/07/2019–31/01/2021)*	% change
Total visits	452	189	+139.2%
Average visit duration	4min24s	2min43s	+62%
Page views	2152	714	+201.4%
Unique page views	1477	500	+195.4%
Searches	128	30	+326.7%
Unique search keywords	46	13	+253.8%
Actions per visit (page views, downloads, outlinks, searches)	5.3	4.1	+29.3%
Max. actions in one visit	75	25	+200%
Visits by continent			
*Europe*	412	185	+122.7%
*North America*	19	3	+533.3%
*Asia*	18	0	—
*South America*	2	1	+100%
*Africa*	1	0	—

## Discussion

The redevelopment of the Translational Data Catalog in line with FAIR principles, has generated marked improvements across all aspects of FAIR for the IMI projects and beyond. Supplementary Table 1 provides an overview of how the Data Catalog addresses each specific FAIR principle.

While the development of a new catalogue may appear to not be entirely consistent with FAIR best practice, the unique focus of this catalogue on IMI projects and datasets, which are not systematically submitted to or indexed in other resources, clearly demonstrates the utility of the resource. By providing persistent project metadata for all IMI projects, we implement a mitigation strategy for information loss resulting from the closure of the projects’ own websites following the end of their IMI funding. Notable examples of projects benefiting from this include IMIDIA (http://www.imi.europa.eu/projects-results/project-factsheets/imidia), DIRECT (http://www.imi.europa.eu/projects-results/project-factsheets/direct), PRECISEADS (http://www.imi.europa.eu/projects-results/project-factsheets/precisesads) and SAFE-T (http://www.imi.europa.eu/projects-results/project-factsheets/safe-t). All of these projects were part of IMI1 and had their project websites retired in recent months, leading to the loss of valuable information. Summary metadata indicating the types of data and experiments performed as part of these projects is however retained in the Data Catalog.

Thanks to the incorporation of Bioschemas markup in the Catalog, the Catalog entries for most projects are now found on the first page of search results in Google, as well as returning top or even direct hits in Google Dataset Search. This improvement in **Findability** of project metadata is a great asset for any project that cannot submit data to public repositories for confidentiality reasons. Bioschemas’ and the DATS model’s compatibility with DCAT and SDO furthermore allows Data Catalog metadata to be indexed in other meta-catalogues, thus further improving findability.

The streamlining of data access requests via the Data Catalog through the encoding of data use conditions using DUO codes increases the **Accessibility** of project data for end-users. Machine-readable data use conditions, encoded in a standardised format using DUO are a key component of FAIR data resources and facilitate the implementation of automatic decision trees to direct users to the correct data access route without the need for unnecessary human intervention. Rather than having to navigate complex data access request systems, users will eventually be able to gain access to various types of datasets via a single point of entry in the Data Catalog.

By adopting the DATS model, an existing community standard that is interoperable with other standards such as DCAT and Bioschemas, we increased the **Interoperability** of Data Catalog metadata with other resources. Users are able to download DATS-compliant metadata and directly integrate it into any DATS-compliant resource.

Making all IMI projects discoverable in a centralised resource using a shared and curated data representation greatly increases the potential for data **Reuse**, despite the very diverse nature of the different projects. By carefully curating keywords for experiment types, data types and other concepts, and underpinning them with ontology annotations, users are able to discover otherwise unrelated projects or datasets that match a set of common parameters.

As a result of these development and curation efforts, we increased the total number of entries from 77 to 356, including a rise from 67 public datasets to 185 (curated and used in previous IMI projects), and increasing the number of IMI projects from 10 to 186.

Ongoing curation efforts aim to constantly expand and improve the information presented in the Data Catalog, for example by expanding the ontology annotations of the existing metadata. These annotations will in turn be leveraged by the Data Catalog’s search functionality through semantic query expansion. We also intend to continue indexing project metadata from IMI’s successor, the IHI.

One major challenge that it is not directly within the control of the Data Catalog to address is the lack of consistent data deposition, which is common to many IMI projects. While the Data Catalog’s study and dataset metadata helps with maintaining a record of the data’s existence, data that is not submitted to some form of repositories, whether institutional, generic or domain-specific, open- or restricted-access, risks being lost after the end of the project for which it was generated. Furthermore, while unpublished data may remain available from the data owner on request, the Data Catalog requires explicit consent from data owners to publish contact information for datasets due to GDPR. Unlike central project contacts, which are also published on the IMI website and whose consent has therefore already been established in this context, data owners can be much harder to track down to obtain consent. Data owners may also be reluctant to be publicly listed as dataset contacts if data-sharing modalities such as data access committees (DACs) have not been properly defined during the lifetime of the project. Ongoing work within ELIXIR Luxembourg seeks to address this through the provision of a comprehensive data type-agnostic, secure and GDPR-compliant data hosting service.

**Fig. 3:**
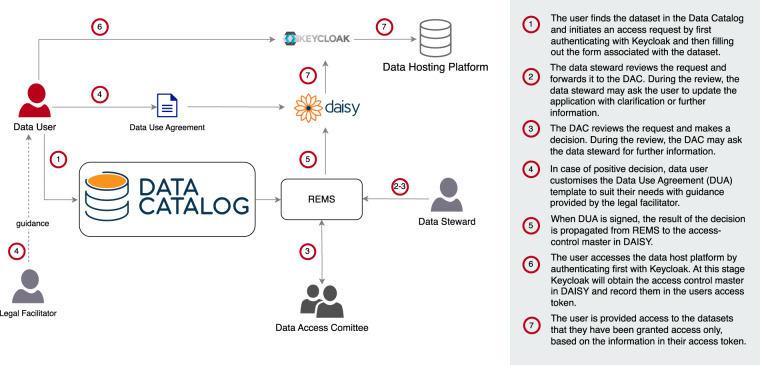
Architecture diagram for the ELIXIR Luxembourg data ecosystem.

These efforts include a data import pipeline to facilitate the import of new information in the Data Catalog directly from a centralised, multi-purpose user submission system based at ELIXIR Luxembourg. In this context, the Data Catalog acts as the public-facing portal that allows discovery of available datasets but also as the gateway for users to request access to these datasets in a secure and managed fashion, as illustrated in Fig. 3. The data hosting and related curation efforts are sustained by the long-term funding of ELIXIR Luxembourg, ensuring the availability of the Data Catalog beyond the funding of the FAIRplus consortium and the IMI. More generally, development efforts on the Data Catalog will continue to address emerging user needs and add new functionalities, with ELIXIR Luxembourg being committed to supporting the long-term sustainability of this invaluable resource.

To conclude, the Data Catalog is a unique resource that cuts across data and experiment types and provides a standardised, curated representation of project- and data-level metadata for a diverse range of projects. It implements the FAIR principles in its design as well as improves the FAIRness of the metadata it presents. Continued development and curation will seek to further the status of the Data Catalog as a fully FAIR-compliant, mature resource of great value to the scientific community.

## Methods

### Model development

We extended the DATS model to improve its applicability to a wide range of data types in the field of translational research. The original DATS model was centred around the generic core concept of a “Dataset”, an entity that covers technical aspects such as licensing, data types and distributions. The Dataset is produced by or is the input or output of a “Study”, which contains elements that are specific to life, environmental and biomedical science domains, and which models experimental processes, cohorts and protocol information. To meet the requirements of project-generated datasets, the DATS model was extended to include the third core concept of “Project”, covering general information such as title, publications, funding and contributors. This effectively created the concept triangle shown in Fig. 1.

In Data Catalog DATS, “Project” is the core part of any entry. Every study and dataset is expected to belong to a project. Each project can contain any number of studies, which in turn can be linked to any number of datasets. Datasets can also be linked directly to a project if no study-related information is available.

Each of the core data objects in DATS contains a set of sub-objects, which in turn contain further sub-objects, down to the lowest unitary object (which contains no further objects), which is the “Annotation”. An “Annotation” consists of just two key-value pairs, the “value” and, optionally, the “valueIRI”, designed to capture the Internationalized Resource Identifier (IRI) of an ontology term contextualising the free text “value”. Due to this nested object structure, DATS can be quite opaque to parse for the human reader but allows for easier programmatic processing of the objects. A full overview of the DATS schema can be found on the DataTagSuite Github repository (see Data & Software availability statement).

In order to remain lightweight and flexible, the original DATS model included very few core properties. Instead, it provided the option to add additional information for any entity via ad-hoc key-value pairs within an entity called “extraProperties”. During the review of the existing content of the Data Catalog, we identified a number of commonly recurring properties, such as “phone number” for project contributors and “project website” for projects, which we decided to include in the model as permanent properties. Table 3 provides an overview of the core as well as recommended properties of each DATS object.

**Table 3:** Core properties of the Data Catalog Data Model.

Data object	Core properties	Recommended properties
PROJECT	title, types, projectLeads	description, acronym, start/end date, funding, projectWebsite
STUDY	name	description, studyGroups, characteristics
DATASET	title, types, creators	description, creation date, version, dataStandard, types, dimensions, isAbout, licenses

Each of the three main data objects - Project, Study and Dataset - has a set of core properties that are required for any object instance to be valid, as well as a set of recommended properties that are not required for formal validation but represent valuable contextual metadata.

While we aim to keep the adapted DATS model relatively stable in order to maximise compatibility with other resources, we also regularly review incoming information regarding new data types to identify recurring information that would benefit from formal integration into the model, including semantic definition via JSON-LD contexts.

Briefly, JSON-LD contexts link the concepts in a JSON document to concept definitions in external standards or ontologies. The DATS model includes context files with mappings to SDO, DCAT and OBO Foundry^7^ ontologies, aiming to provide at least one context mapping for every property in the model, with some properties mapped to multiple equivalent external concepts. This greatly improves the semantic interoperability of data encoded in the model.

### Web portal development

The Data Catalog user portal web application was redeveloped with extended search facilities as well as a more appealing and intuitive user interface, improving the user experience. The three concepts from the core model are presented as separate facets in the user interface, allowing the user to choose which aspect they are most interested in. Concepts are, however, also fully interlinked to allow users to easily move between them and search across them. As part of the redevelopment, we also reviewed the properties displayed in the original Data Catalog and adapted these to allow the inclusion of a more diverse range of studies and datasets.

**Fig. 4:**
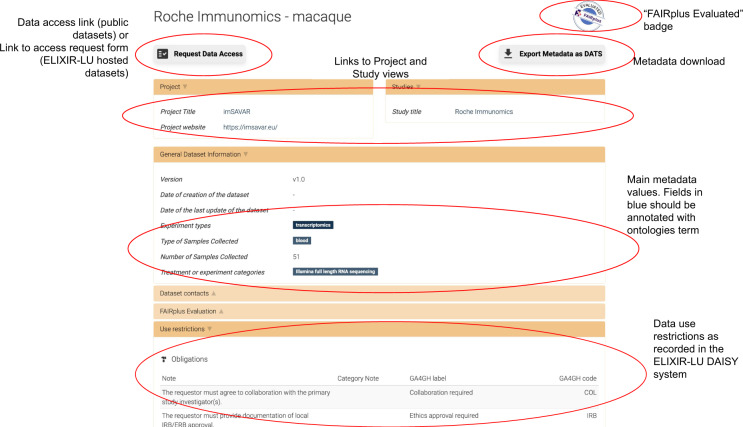
Illustration of the new Data Catalog user interface. This figure shows the dataset page for one of the Roche Immunomics datasets, part of the imSAVAR project (https://www.imi.europa.eu/projects-results/project-factsheets/imsavar), including the linking panels for the Project and Study pages, the general information panel with various metadata values including Experiment Types and Sample Types, and the Data Use Restrictions panel. The page also highlights the FAIRplus Evaluated badge.

The Data Catalog stores and indexes the metadata using Solr. The backend is a Python application using Flask as a web framework. The user interface is developed using HTML, CSS and jQuery. A few facets are pre-defined (data types, disease, keywords, samples types) to provide rapid filters and it is possible to change the defaults or add more by configuration. A search function is provided to enable search on a selection of fields depending on the entity type (e.g. description, keywords, title and types for projects). This selection of fields used for the main search can also be changed through configuration. It is also possible to assign weights to each of those fields. To highlight the projects evaluated and FAIRified by FAIRplus, a highlight function is implemented, as shown in Figs. 2, 4.

The addition of data access or request functionality and downloadable metadata in JSON format are further innovations designed to address FAIR requirements.

### Bioschemas integration

**Fig. 5:**
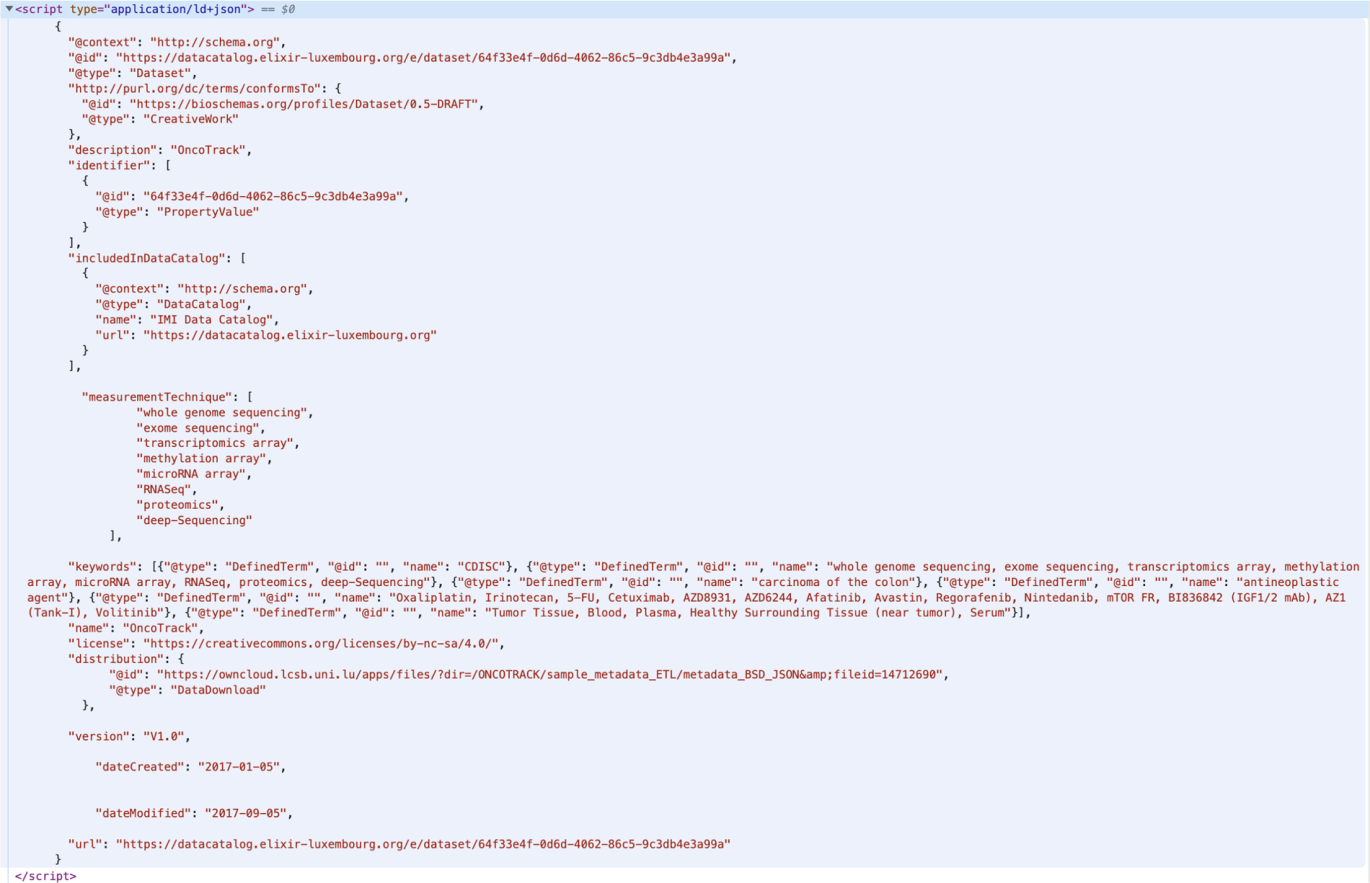
An example of the page headers showing the Bioschemas integration for the OncoTrack dataset. The header snippet shows the instantiation of the Bioschemas Dataset profile for the ONCOTRACK (http://www.imi.europa.eu/projects-results/project-factsheets/onco-track) dataset, found at https://datacatalog.elixir-luxembourg.org/e/dataset/64f33e4f-0d6d-4062-86c5-9c3db4e3a99a.

As part of the web portal redevelopment, we integrate markup for several Bioschemas profiles in the Data Catalog pages. In the first instance, we made use of the “DataCatalog” profile, which can be found on every page of the IMI Catalog, and the “Dataset” profile, which is built dynamically for each “Dataset” page from the human-readable content. An example of the markup is shown in Fig. 5.

At present, there is no Bioschemas-specific profile for “Project” and the “Study” profile is still under development. We are working with the Bioschemas community to drive the expansion of suitable profiles by providing compelling use cases for both.

### Data compilation

Data Catalog data is gathered from a number of sources. During the redevelopment process, all the data from the first version of the Data Catalog was migrated to the new DATS model. Project-level metadata for all IMI projects was imported from the IMI Project factsheets using an automatic web scraping script. Study- and dataset-level metadata for selected projects was collected directly from the study authors or from publications and data submitted to publicly available repositories. For interaction with study authors, we created a simplified version of the DATS model in an Excel spreadsheet that allows the collection of all the key values of interest without the need to understand the complexities of the data model.

Finally, we are working on an end-to-end pipeline integrating a range of resources hosted at ELIXIR Luxembourg, including DAISY (DAta Information SYstem), an open-source web application that allows biomedical research institutions to map their data and data flows in accordance with General Data Protection Regulation (GDPR) requirements and our Atlas servers for data analysis. When fully operational, the pipeline will present a single data entry interface for users to submit all relevant data and metadata for DAISY, Atlas and the Data Catalog. The pipeline will then distribute all records to the appropriate resources in line with any data restrictions and embargoes specified by the submitter. It will for example be possible for Data Catalog metadata to be held as part of an access-controlled record in DAISY during a multi-stage submission, until such a time as the submitter is ready to release some or all of the metadata to the Data Catalog.

### Version control

All metadata included in the Data Catalog is kept in a dedicated internal Gitlab repository, separate from the infrastructure code. While there are no formal batch data releases to the Data Catalog, with records being pushed to production as they become available, Gitlab’s built-in version control mechanisms make it possible to keep track of changes to records. At present, only the latest version for each Catalog entry is shown. We are investigating the possibility of making previous versions accessible but have not yet identified a compelling use case to prioritise this feature.

## Supplementary information

Supplementary Table 1

## Data Availability

The Data Catalog is available at https://datacatalog.elixir-luxembourg.org/.

## Code availability

All Data Catalog code is available in a dedicated repository of the FAIRplus Github organisation, at https://github.com/FAIRplus/imi-data-catalogue. The repository includes full documentation on how to deploy a stand-alone version of the Data Catalog.

The DATS model is available on Github at https://github.com/datatagsuite/schema.
