# Supplementary material for: The Translational Data Catalog - discoverable biomedical datasets
Source: Sci Data. 2023 Jul 20;10:470. doi: 10.1038/s41597-023-02258-0 (PMC10359386; doi:10.1038/s41597-023-02258-0)
Supplement: Supplementary file 1 — Supplementary Table 1 [file 41597_2023_2258_MOESM1_ESM.pdf]

Supplementary Table 1

This table contains the full list of 15 FAIR principles and sub-principles as generated by Wilkinson et al., and how the Translational Data Catalog conforms to each principle.

Principle	Description	Applied in the Data Catalog
F1	(Meta)data are assigned a globally unique and persistent identifier	Every entry in the Data Catalog is assigned a Universally Unique IDentifier (UUID), which as per our curation policy must remain public with at least minimal metadata once it has been released.
F2	Data are described with rich metadata (defined by R1 below)	While there is no formal definition of “rich metadata”, the authors believe that DATS model more than adequately fulfils this requirement.
F3	Metadata clearly and explicitly include the identifier of the data they describe	Every record in the Data Catalog for which data is publicly available includes both the identifier and a direct link to the data
F4	(Meta)data are registered or indexed in a searchable resource	Data Catalog metadata is indexed in Google (incl Google Dataset Search) and other major search engines through the use of Bioschemas markup
A1	(Meta)data are retrievable by their identifier using a standardised communications protocol	The entire Data Catalog infrastructure uses HTTPS
A1.1	The protocol is open, free, and universally implementable	See A1
A1.2	The protocol allows for an authentication and authorisation procedure, where necessary	See A1
A2	Metadata are accessible, even when the data are no longer available	The Data Catalog only hosts metadata, including metadata for IMI project datasets such as data from the IMIDIA project, which is no longer available. In addition, our curation policy requires minimum metadata such as the UUID and title/acronym of an entry to remain available even if other metadata has been removed.

I1	(Meta)data use a formal, accessible, shared, and broadly applicable language for knowledge representation.	All Data Catalog metadata is available in JSON-LD format.
I2	(Meta)data use vocabularies that follow FAIR principles	While we cannot guarantee the full FAIR compliance of every ontology used in the Data Catalog, we believe that all ontologies used mostly follow the FAIR principles, including NCIt, ChEBI, SIO, OBI, MONDO and EDAM.
I3	(Meta)data include qualified references to other (meta)data	All Data Catalog metadata is available in JSON-LD format, which effectively addresses this requirement
R1	(Meta)data are richly described with a plurality of accurate and relevant attributes	See F2
R1.1	(Meta)data are released with a clear and accessible data usage license	All Data Catalog metadata is released under CC BY-NC-SA 4.0. This is encoded in the Bioschemas markup in the header of every page.
R1.2	(Meta)data are associated with detailed provenance	Where available, contact details are included with each Data Catalog entry. In addition, the DATS model is able to encode full provenance information although this is not available for most of our metadata due to its origins.
R1.3	(Meta)data meet domain-relevant community standards	All Data Catalog metadata conforms to the DATS model, which fulfils this requirement in itself and is also compatible with other community standards such as DCAT and SDO.
